# Supplementary material for: Statistics of pathogenic bacteria in the search of host cells
Source: Nat Commun. 2021 Mar 31;12:1990. doi: 10.1038/s41467-021-22156-6 (PMC8012381; doi:10.1038/s41467-021-22156-6)
Supplement: Supplementary file 1 — Supplementary Information [file 41467_2021_22156_MOESM1_ESM.pdf]

Supplementary Information
Statistics of pathogenic bacteria in the search of host cells

Stefan Otte,[1,2] Emiliano Perez Ipiña,[3,1,2] Rodolphe Pontier-Bres,[4,2] Dorota Czerucka,[4,2] and Fernando Peruani[1,2,5]

[1] *Université Côte d'Azur, Laboratoire J.A. Dieudonné, UMR 7351 CNRS, Parc Valrose, Nice F-06108, France*

[2] *LIA ROPSE, Laboratoire International Associé Université Côte d'Azur - Centre Scientifique de Monaco*

[3] *Department of Physics & Astronomy, Johns Hopkins University, Baltimore, Maryland 21218, USA*

[4] *Centre Scientifique de Monaco (CSM), 8 Quai Antoine 1er, Monaco 98000, Principality of Monaco*

[5] *Laboratoire de Pysique Théorique et Modélisation, UMR 8089, CY Cergy Paris Université, 95302 Cergy-Pontoise, France*

SUPPLEMENTARY NOTE 1: DERIVATION OF $\langle \mathbf{x}(t)\rangle$, $\langle \mathbf{x}^2(t)\rangle$ AND DIFFUSION COEFFICIENT D

The probability distribution $p(\mathbf{x}, t)$ of finding a chiral active particle with active fluctuations – defined by Eqs. (3) – at position $\mathbf{x}$ at time t exhibits a rather complex dynamics: its average evolves in time as well as its second moment. To circumvent the complex problem of computing $p(\mathbf{x}, t)$, we will limit ourselves to calculate directly its first moment (the average position) and second moment (mean-squared displacement), which are in fact the quantities of interest for us; from the second moment we obtain the diffusion coefficient D. This shortcut is possible because we have intentionally defined a model for chiral active walkers where the dynamics of θ decouples of that of v. With this in mind, we recall that by definition $\mathbf{x}(t) = \int_0^t \dot{\mathbf{x}}(t')\mathrm{d}t'$. By taking the average value, we obtain:

$$\langle \mathbf{x}(t)\rangle = \langle \int_0^t \dot{\mathbf{x}}(t')\mathrm{d}t'\rangle = \int_0^t \langle \dot{\mathbf{x}}(t')\rangle \mathrm{d}t' = \int_0^t \langle v(t')\mathbf{e}(\theta(t'))\rangle \mathrm{d}t' \,, \tag{1}$$

where we have used explicitly that defines $\dot{\mathbf{x}}(t) = v(t)\mathbf{e}(\theta(t))$. It is evident that $\langle \dot{\mathbf{x}}(t)\rangle = \langle v(t)\mathbf{e}(\theta(t))\rangle = \langle v(t)\rangle\langle \mathbf{e}(\theta(t))\rangle$ provided the requested independence between v and θ. The problem reduces then to compute $p(v, t)$ and $p(\theta, t)$, which can be done by solving the Fokker-Planck equations associated with the eqs. (3c) and (3b), respectively. Along similar lines, we can obtain an expression for the second moment of $p(\mathbf{x}, t)$ by integrating:

$$\langle \mathbf{x}^2(t)\rangle = \int_0^t \int_0^t \langle \dot{\mathbf{x}}(t')\dot{\mathbf{x}}(t'')\rangle \mathrm{d}t'\mathrm{d}t'' \,, \tag{2}$$

expression that is often referred to as Taylor-Kubo formula. Using the independence of v and θ, it is easy to show that $\langle \dot{\mathbf{x}}(t')\dot{\mathbf{x}}(t'')\rangle = \langle v(t')\, v(t'')\rangle\langle \cos(\theta(t') - \theta(t''))\rangle$. The rest of the section is organized as follows: we first provide expressions for autocorrelation functions of $\mathbf{e}(\theta(t))$ and $v(t)$, to finally use these expressions to compute $\langle \mathbf{x}(t)\rangle$, $\langle \mathbf{x}^2(t)\rangle$, and D_{eff}.

The Langevin Eq. (3b) for the moving direction $\theta(t)$ is independent from other variables and can be treated analytically. The autocorrelation function of the director $\mathbf{e}(t) = (\cos\theta(t), \sin\theta(t))$ can be written as

$$B(\,t\,) = \langle \frac{\mathbf{V}(t')}{u(t')}\frac{\mathbf{V}(t' +\, t\,)}{u(t' +\, t\,)}\rangle = \langle \mathbf{e}(t')\mathbf{e}(t' +\, t)\rangle = \mathrm{e}^{-D_\theta\, t}\cos(\Omega\, t\,) \,. \tag{3}$$

$$\begin{aligned}\langle \mathbf{e}(t)\mathbf{e}(t')\rangle &= \langle \cos(\theta(t) - \theta(t'))\rangle \\ &= \iint_0^{2\pi} \cos(\theta - \theta')\, p(\theta, t, \theta', t')\mathrm{d}\theta\mathrm{d}\theta' \,.\end{aligned} \tag{4}$$

We use the fact that $p(\theta, t, \theta', t') = p(\theta, t|\theta', t')\cdot p(\theta', t')$ to rewrite Supplementary Eq. (4). Thus, in order to evaluate the integral we need to compute the conditional probability distribution. This probability is obtained by solving the Fokker-Planck equation corresponding to the Langevin (3b) using as initial condition $p(\theta, t = 0) = \delta(\theta - \theta')$. The corresponding Fokker-Planck equation reads:

$$\partial_t\, p(\theta, t) = -\Omega\partial_\theta\, p(\theta, t) + D_\theta\partial_\theta^2\, p(\theta, t').$$

Using the ansatz $p(\theta,t)=T(t)A(\theta)$, the periodic nature of θ and the initial condition, the final conditional probability becomes,

$$\begin{aligned} p(\theta,t|\theta',t') &= \sum_{n=-\infty}^{\infty}\left(\frac{1}{2\pi}\mathrm{e}^{-in\theta'}\right)\mathrm{e}^{-n^2D_\theta|t-t'|-i\Omega n|t-t'|+in\theta} \\ &= \frac{1}{2\pi}+\frac{1}{\pi}\sum_{n=1}^{\infty}\mathrm{e}^{-n^2D_\theta|t-t'|}\cos\left(n\Omega|t-t'|-n(\theta-\theta')\right), \end{aligned} \tag{5}$$

where $|t-t'|>0$ is the time elapsed between observing the angle θ after observing the angle θ' at t'. For $t\to\infty$, the steady-state distribution is obtained:

$$p(\theta)=\frac{1}{2\pi}, \tag{6}$$

which is a uniform distribution for the angle $\theta\in[0,2\pi)$. Inserting Supplementary Eq. (5) and Supplementary Eq. (6) in Supplementary Eq. (4), the correlation function of the moving direction can be calculated:

$$\langle\mathbf{e}(t)\mathbf{e}(t')\rangle=\mathrm{e}^{-D_\theta|t-t'|}\cos\left(\Omega|t-t'|\right). \tag{7}$$

The correlation function of the director is an oscillating cosine function where the oscillation frequency is given by the parameter Ω. The amplitude of the cosine is a decaying exponential with a decay rate given by the parameter D_θ.

The Langevin Eq. (3c) for the speed v is also independent from other variables and defines a standard *Ornstein-Uhlenbeck* process. The instantaneous speed correlation function is defined as

$$\langle v(t)v(t')\rangle=\int_0^\infty\int_0^\infty vv'p(v,t,v',t')\mathrm{d}v\mathrm{d}v'. \tag{8}$$

To evaluate Supplementary Eq. (8), we use that $p(v,t,v',t')=p(v,t|v',t')\,p(v',t')$. The expressions for these distribution functions are obtained from the associated Fokker-Planck equation of (3c) [1]:

$$\partial_t p(v,t)=k_v\partial_v\left[(v-\bar{v})\,p(v,t)\right]+D_v\partial_v^2p(v,t). \tag{9}$$

Using the Fokker-Planck equation above with different initial conditions, we find,

$$\begin{aligned} p(v,t|v',t'=0) &= \frac{1}{\sqrt{2\pi\frac{D_v}{k_v}\left(1-\mathrm{e}^{-2k_vt}\right)}} \\ &\quad\times\exp\left[\frac{-\left((v-\bar{v})-(v'-\bar{v})\mathrm{e}^{-k_vt}\right)^2}{2\frac{D_v}{k_v}\left(1-\mathrm{e}^{-2k_vt}\right)}\right]. \end{aligned} \tag{10}$$

For $t\to\infty$, one obtains the steady-state distribution

$$p(v)=\frac{1}{\sqrt{2\pi\frac{D_v}{k_v}}}\exp\left[\frac{-(v-\bar{v})^2}{2\frac{D_v}{k_v}}\right] \tag{11}$$

which is a normal distribution, centered at $\bar{v}$ with variance D_v/k_v. For $p(v',t')$, i.e. the probability of the speed v' at a random time t' in Supplementary Eq. (8), one can now use the steady state distribution, Supplementary Eq. (11). Furthermore, inserting Supplementary Eq. (10) for the conditional probability $p(v,t|v',t')$ yields for the instantaneous speed autocorrelation function,

$$\langle v(t)v(t')\rangle=\bar{v}^2+\frac{D_v}{k_v}\cdot\mathrm{e}^{-k_v|t-t'|}. \tag{12}$$

This result could alternatively be obtained using directly the Langevin (3c) as well as properties of the noise $\xi_v(t)$ as in [2]. The normalized speed autocorrelation function $A(\tilde{t}\,)$ can now be defined as

$$A(\tilde{t}\,):=\frac{\langle v(\tilde{t})v(0)\rangle-\bar{v}^2}{(D_v/k_v)}=\mathrm{e}^{-k_v\tilde{t}} \tag{13}$$

where $\tilde{t} = |t - t'|$. It is a decaying exponential function, where the decay rate is given by the parameter k_v. Thus, the parameter k_v is indeed the correlation timescale of the speed v.

Given the distributions $p(v,t)$ and $p(\theta,t)$ in hand, it is straight forward to derive the full expression of the average position of a particle staring at point $\mathbf{x}_0 = 0$ with speed v_0 and direction θ_0,

$$\begin{aligned}\langle \mathbf{x}(t)\rangle = &\frac{\bar{v}}{D_\theta^2+\Omega^2}\left[D_\theta \mathbf{e}(\theta_0) + \Omega \mathbf{e}_\perp(\theta_0) - \mathrm{e}^{-D_\theta t}\left(D_\theta \mathbf{e}(\theta_0+\Omega t) + \Omega \mathbf{e}_\perp(\theta_0+\Omega t)\right)\right] \\ &+ \frac{v_0-\bar{v}}{(D_\theta+k_v)^2+\Omega^2}\left[(D_\theta+k_v)\,\mathbf{e}(\theta_0) + \Omega\mathbf{e}_\perp(\theta_0) - \mathrm{e}^{-(D_\theta+k_v)t}\left((D_\theta+k_v)\,\mathbf{e}(\theta_0+\Omega t) + \Omega\mathbf{e}_\perp(\theta_0+\Omega t)\right)\right] \quad (14)\end{aligned}$$

where $\mathbf{e}(\theta) = (\cos\theta, \sin\theta)$ and $\mathbf{e}_\perp(\theta) = (-\sin\theta, \cos\theta)$. Notice that the nontrivial temporal evolution of the average position is therefore an inward logarithmic spiral for $D_\theta > 0$ that converges asymptotically to a constant value, while for $D_\theta = 0$ it becomes outward spiral that asymptotically converges to a limit cycle.

To calculate the second moment, we observe first that:

$$\langle \dot{\mathbf{x}}(t)\dot{\mathbf{x}}(t')\rangle = \langle v(t)v(t')\cos(\theta(t)-\theta(t'))\rangle \quad (15)$$

$$= \int vv'\cos(\theta-\theta')\,p(v,t,v',t')\cdot p(\theta,t,\theta',t')\mathrm{d}v\mathrm{d}v'\mathrm{d}\theta\mathrm{d}\theta' \quad (16)$$

where we use that $p(\theta,v,t,\theta',v',t') = p(v,t,v',t')\cdot p(\theta,t,\theta',t')$. By performing the integrals, we find that the velocity autocorrelation function becomes,

$$\begin{aligned}\langle \dot{\mathbf{x}}(t)\dot{\mathbf{x}}(t')\rangle &= \langle v(t)v(t')\rangle \cdot \langle \mathbf{e}(t)\mathbf{e}'(t')\rangle \\ &= \left(\bar{v}^2 + \frac{D_v}{k_v}\cdot \mathrm{e}^{-k_v|t-t'|}\right)\mathrm{e}^{-D_\theta|t-t'|}\cos(\Omega|t-t'|) \quad (17)\\ &= f\left(\bar{v}^2,\, D_\theta,\, \Omega,\, t,\, t'\right) + f\big((D_v/k_v),\, (D_\theta+k_v),\, \Omega,\, t,\, t'\big)\end{aligned}$$

where the function f is defined as $f(a,\,b,\,c,\,t,\,t') := a\,\mathrm{e}^{-b|t-t'|}\cos(c|t-t'|)$.

Thus, the velocity autocorrelation function becomes a sum of two terms of the same functional form. Now, the calculation of the second moment requires evaluating

$$\begin{aligned}F(a,b,c,t) &:= \iint_0^t f(a,\,b,\,c,\,t',\,t'')\mathrm{d}t'\mathrm{d}t'' \\ &= 2a\left[\frac{b}{b^2+c^2}t + \frac{(b^2-c^2)\left(\cos(ct)\mathrm{e}^{-bt}-1\right) + 2bc\sin(ct)\mathrm{e}^{-bt}}{(b^2+c^2)^2}\right] \quad (18)\end{aligned}$$

Combining Supplementary Eq. (2), Supplementary Eq. (17) and Supplementary Eq. (18) yields the full expression for the second moment:

$$\begin{aligned}\langle \mathbf{x}^2(t)\rangle = &2\bar{v}^2\left[\frac{D_\theta}{D_\theta^2+\Omega^2}t + \frac{(D_\theta^2-\Omega^2)\left(\cos(\Omega t)e^{-D_\theta t}-1\right) + 2D_\theta\Omega\sin(\Omega t)e^{-D_\theta t}}{(D_\theta^2+\Omega^2)^2}\right] \\ &+ 2\frac{D_v}{k_v}\left[\frac{(D_\theta+k_v)}{(D_\theta+k_v)^2+\Omega^2}t + \frac{\left((D_\theta+k_v)^2-\Omega^2\right)\left(\cos(\Omega t)e^{-(D_\theta+k_v)t}-1\right) + 2\,(D_\theta+k_v)\,\Omega\sin(\Omega t)e^{-(D_\theta+k_v)t}}{\left((D_\theta+k_v)^2+\Omega^2\right)^2}\right]. \quad (19)\end{aligned}$$

Finally, using the above results of Supplementary Eqs. (14) and (19), we compute the full expression for the diffusion coefficient,

$$\begin{aligned}D &= \lim_{t\to\infty}\frac{\langle \mathbf{x}^2(t)\rangle - \langle \mathbf{x}(t)\rangle^2}{4t} \\ &= \frac{\bar{v}^2 D_\theta}{2\,(D_\theta^2+\Omega^2)} + \frac{D_v}{2k_v}\frac{(k_v+D_\theta)}{\left((k_v+D_\theta)^2+\Omega^2\right)}. \quad (20)\end{aligned}$$

This Supplementary Note is about the derivation of the main mathematical expressions supporting this study. Note that all Supplementary Eqs. are not mentioned in the main text, except for Supplementary Eqs. (7), (13) and (20), which correspond to Eqs. (2), (1) and (5) in the main text, respectively.

SUPPLEMENTARY NOTE 2: PARAMETER ESTIMATION

Analysis of trajectories

From the tracking, a set of points $\{\mathbf{x}_i\}$ with $\mathbf{x}_i = (x(t_i), y(t_i))$ at times $t_i = i \cdot \Delta t$ for $i = 1, ..., N$ time steps was obtained for each bacterium, ($\Delta t =$(35fps)$^{-1}$). The parameter values for each trajectory were obtained as detailed below. All fittings to the expressions derived below were done using the Levenberg-Marquardt nonlinear least-squares algorithm [3] as implemented in the scipy ecosystem [4]. If not stated otherwise, errors of the parameter values are one standard-deviation errors (SE), calculated from the covariance matrix given by the algorithm and exploiting the reduced χ^2, as described in [3]. Errors of quantities calculated from several parameter values, e.g. the effective diffusion coefficient, are obtained by Gaussian propagation of error. The positional localization errors are given by the automatic tracking method. To locate the position of a bacterium, the detector algorithm applies a Laplacian of Gaussian filter (LoG) and then searches for the circle of diameter equal to the size of the bacterium with maximum intensity. According to [5], this detector method yields to sub-pixel localization errors, $\delta \leq 0.3\mu$m, (SNR>3). This is consistent with a rough estimate of the positional error, where a circle of the size of the bacterium is placed randomly over the bacterium body. In this case, it would be reasonable to expect that the circle includes at least half of the bacterium, leading to an error between the center of the circle and the true bacterium center of mass of $\delta = (l/2)/\sqrt{12}$. Considering a bacterium of size $l = 2\mu$m, then $\delta = (l/2)/\sqrt{12} \approx 0.29\mu$m for our rough estimation, which is very close to the pixel size. To be conservative, we consider the positional error to be as large as 1 px, *i.e.* $\delta = 0.3\mu$m, which leads to speed measurement error (using two consecutive frames) of $\pm 10\mu$m/s.

The characterization of each experimental trajectory involves the estimation of the 5 model parameters: Ω, D_θ, $\bar{v}$, D_v, and k_v. It is important to notice that experimentally we only estimates the position of the center of mass of the bacterium every $\Delta t = 0.03$ s – with Δt the time between two consecutive frames – but not to the instantaneous speed and moving direction, while Eqs. (3) logically deal with the temporal evolution of instantaneous and exact variables. To circumvent this problem, we use the theory to estimate predictions on the statistics of discrete displacements. From the data, we construct $\Delta\mathbf{x}_{i,n} = (\Delta x_{i,n}, \Delta y_{i,n}) = (x(t_i + n\Delta t) - x(t_i), y(t_i + n\Delta t) - y(t_i))$, where t_i refers to the time associated to frame i, and n is a non-negative integer that we choose such that Ω^{-1}, $D_\theta^{-1} \gg n\Delta t$, a condition that is satisfied for $n \leq 7$. In this way, we obtain an average velocity vector $\mathbf{V}_{i,n} = \Delta\mathbf{x}_{i,n}/(n\Delta t)$, from which we extract an average speed $u_{i,n} = \sqrt{\Delta x_{i,n}^2 + \Delta y_{i,n}^2}/(n\Delta t) = d_{i,n}/(n\Delta t)$. Considering there are no experimental errors and our estimates are exact, we can connect the average speed with the theory by $u_{i,n} = (n\Delta t)^{-1} \int_{t_i}^{t_i+n\Delta t} v(s)ds$. As shown in Fig. 1(f), from the maximum of the distribution of $u(t_i)$ we estimate $\bar{v}$. For the width of this distribution we get:

$$\sigma_{u,n}^2 = \langle (u_{i,n} - \bar{v})^2 \rangle_i \approx 2\frac{D_v}{k_v^3 [n\Delta t]^2} \phi(k_v n\Delta t)\,, \tag{21}$$

where $\phi(z) = e^{-z} + z - 1$ and $\langle ... \rangle_i$ denotes average over i. Now, as stated before, expression (21) is exact in the case where the positions of the center of mass are known without experimental uncertainty. However, the observed speed fluctuations can have a contribution coming from the positional error made at localizing the bacterium center of mass at each frame. To understand this contribution and to be able to discriminate between intrinsic fluctuations of the studied phenomenon and those coming from experimental errors, it is more convenient to work with the average displacements, $d_{i,n}$ instead of the average speed. Then, we can write the displacement fluctuations as, $\langle (d_{i,n} - \bar{v}n\Delta t)^2 \rangle = 2\frac{D_v}{k_v^3 n\Delta t}\phi(k_v n\Delta t) + 2\delta^2$. Note that this expression gives us a way to distinguish between both sources of fluctuations. While the fluctuations due to the Ornstein–Uhlenbeck process depends on $n\Delta t$, the experimental ones are constant. In the limit of large k_v, the intrinsic fluctuations are proportional to $n\Delta t$, see Supplementary Fig. 1(c). The fact that the measured fluctuations grow linearly with $n\Delta t$ is a proof that the observed speed fluctuations are intrinsic to the bacteria motility and not due to the experimental positional uncertainty. Moreover, from the slope of the curve we can estimate the value of $\frac{D_v}{k_v^2}$, see Supplementary Fig. 1(c). For large values of k_v, the above expression provides us with an estimate for D_v/k_v^2. From the correlations of $u_{i,n}$, defined as:

$$A(j\Delta t) = \frac{\langle (u_{i,n} - \bar{v})\,(u_{i+j,n} - \bar{v}) \rangle_i}{\langle (u_{i,n} - \bar{v})^2 \rangle_i} = e^{-k_v j\Delta t}\,, \tag{22}$$

we obtain an estimate of k_v (Fig. 1(g)). For $k_v \gg \Omega, D_\theta$ the transport properties, *e.g.* the diffusion coefficient, become independent of the actual value of k_v, and depend only on the ratio D_v/k_v^2. Fig. 1 shows in more detail how the model is used to fit the experimental data.

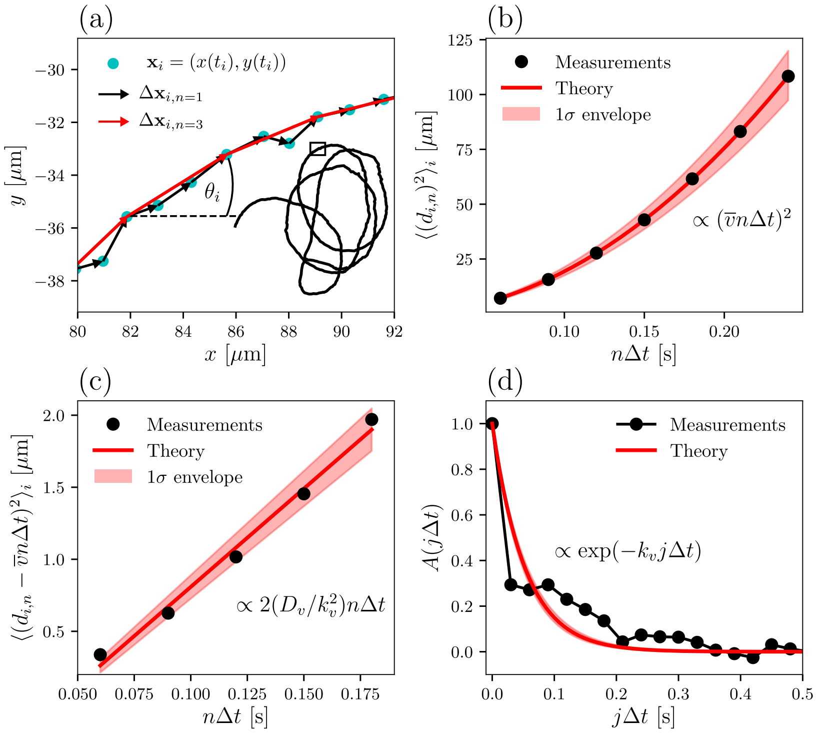

Supplementary Fig. 1: Measurable observables for obtaining the parameters related to the speed. (a): From the measured positions $\mathbf{x}_i$ of the bacteria, displacement vectors $\Delta\mathbf{x}_{i,n}$ can be calculated for different step lengths n. The angle between displacement vector and x-axis is the moving direction θ. The trajectory appears almost straight for a few time steps. (b): The second moment $\langle (d_{i,n})^2 \rangle_i$ for $n = 2, ..., 8$ with $d_{i,n} = \sqrt{\Delta x_{i,n}^2 + \Delta y_{i,n}^2}$ is a quadratic polynomial involving the parameter $\bar{v}$ as a coefficient. (c): The variance $\langle (d_{i,n} - \bar{v}n\Delta t)^2 \rangle_i$ of the displacements yields a quantity which grows linear in n, where the linear coefficient involves the parameter combination (D_v/k_v^2). Error bands in (b) and (c) correspond to SE. (d): The normalized temporal autocorrelation function $A(j\Delta t)$ of the averaged speed is a decaying function, with a timescale k_v^{-1} that can be estimated using the theoretical expression Supplementary Eq. (13).

Lastly, from the (average) correlation of the moving direction:

$$B(j\Delta t) = \langle \frac{\mathbf{V}_{i,n}}{u_{i,n}} . \frac{\mathbf{V}_{i+j,n}}{u_{i+j,n}} \rangle_i = e^{-D_\theta j \Delta t} \cos(\Omega j \Delta t) \ , \tag{23}$$

it is possible to extract the values of Ω and D_θ (Fig. 1(c) and Supplementary Fig. 2). The correlation function for trajectories where the rotation is clockwise (CW) in Supplementary Fig. 2(b) (black curve) is in good agreement with the theoretical correlation function of the director $\langle \mathbf{e}(t)\mathbf{e}(t') \rangle = \mathrm{e}^{-D_\theta |t-t'|} \cos(\Omega |t-t'|)$ for the *instantaneous* angle θ, (red-dashed curve). Hence, fitting of Supplementary Eq. (23) yields an estimate for the model parameters D_θ and Ω. The director correlation function of the straight trajectory is displayed in Fig. 2(c). In this case, $\Omega \approx 0$ by definition of a straight trajectory so the correlation function has no visible dependence on Ω and is simply a decaying exponential. Again, the theoretical expression (red-dashed line) is in good agreement with the measured correlation function. Therefore, in the case of small Ω, only D_θ can be obtained from the correlation function and a different and independent measure for Ω is needed. One possibility exploits the curvature κ of the trajectories. The insets of Supplementary Fig. 2(a) show parts of the straight and CW trajectories, respectively. Both insets also feature a filtered trajectory, obtained by smoothing the $x_i(t_i)$ and $y_i(t_i)$ component of the trajectory separately using a Gaussian filter with standard deviation $\sigma_G = 5$. This filtering eliminates effects of the precessive motion of the bacterium around the average moving direction from the trajectory. For the filtered trajectories, the curvature κ can be calculated geometrically using

$$\kappa(t_i) = -\frac{|\ddot{\mathbf{x}}(t_i) \times \dot{\mathbf{x}}(t_i)|}{|\dot{\mathbf{x}}(t_i)|^3} = \frac{\dot{x}(t_i)\ddot{y}(t_i) - \dot{y}(t_i)\ddot{x}(t_i)}{(\dot{x}^2(t_i) + \dot{y}^2(t_i))^{3/2}} . \tag{24}$$

The curvatures obtained for the straight and CW trajectories are displayed in 2(e) (solid blue and brown curves) as

well as the temporal average $\bar{\kappa} = \langle \kappa(t_i) \rangle_i$ (dashed lines). As expected, $\bar{\kappa}$ for the straight trajectory is closer to zero than for the CW trajectory. Finally, Ω and $\bar{\kappa}$ are geometrically related by $\Omega = \bar{\kappa} \cdot \bar{v}$, such that Ω can be obtained if $\bar{\kappa}$ and the mean speed $\bar{v}$ were measured independently. The two independent ways to obtain the parameter Ω, namely Ω_{Corr} from a fit to the autocorrelation function of the director and Ω_κ from the curvature and the mean speed, should yield equivalent results. Supplementary Fig. 2(f) shows $|\Omega_{Corr}|(|\Omega_\kappa|)$ for all tracked trajectories: As all points fall close to the line $y = x$, both methods give very similar results for Ω. However, note that Ω_{Corr} does not take on values between 0 and 0.25, meaning that the method using the correlation function of the director is not useful for trajectories with a value of Ω in this range. A qualitative distinction between the two cases ($\Omega \approx 0$) and ($\Omega > 0$) can be used to decide the method to fit Ω. While the Fast Fourier Transform of a straight trajectory has a maximum at $\Omega = 0$, the one for the CW trajectory has a maximum at a value $\Omega > 0$ (Supplementary Fig. 2(d)). Then, Ω for an individual trajectory is defined as

$$\Omega := \begin{cases} \Omega_{Corr} & \text{if FFT has a maximum at } \Omega \neq 0 \\ \bar{\kappa} \cdot \bar{v} & \text{else} \end{cases} \tag{25}$$

If $\Omega = \bar{\kappa} \cdot \bar{v}$, the error of Ω is calculated using Gaussian propagation of the errors of $\bar{v}$ and $\bar{\kappa}$. For the error of $\bar{\kappa}$, the standard error of the mean value is used.

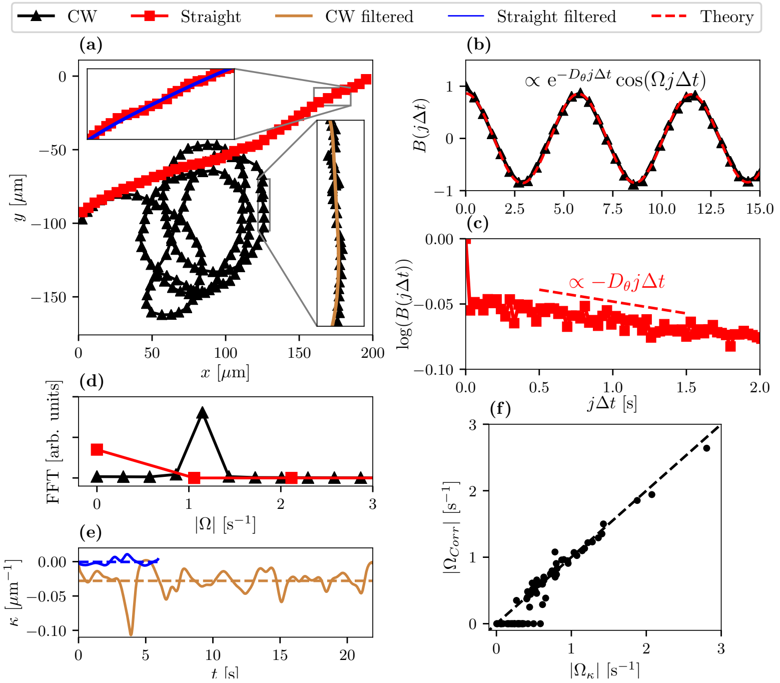

Supplementary Fig. 2: Measurable observables for obtaining the parameters related to the moving direction. (a): Two example trajectories of bacterial motion which show straight (straight, red square) and clockwise (CW, black triangle) motion on the glass surface. The solid blue (straight filtered) and brown (CW filtered) lines correspond to the above trajectories smoothed using a gaussian filter. (b): The autocorrelation function of the director for the CW trajectory agrees well with the theoretical expression (red-dashed line), such that it can be used to obtain both angle-related parameters Ω and D_θ. (c): The same autocorrelation function for the straight trajectory does not depend on the parameter Ω, which is close to 0 in this case, but still shows the exponential decay due to the effect of D_θ. Ω cannot be obtained from the autocorrelation in this case. (d): Fast Fourier transforms of the correlation functions shown in (b), (c). The maxima occur at $\Omega = 0$ for the straight and $\Omega > 0$ for the CW trajectory. This difference can be used as a qualitative measure for "straight" and "non-straight" trajectories. (e): The curvatures κ over time for the filtered trajectories as shown in the insets of (a). The average values (dashed lines) provide an alternative strategy for estimating Ω. (f): Ω_{Corr} obtained from the director autocorrelation function versus Ω_κ (black circles) obtained from the curvature for all trajectories: As the values fall close to the line $y = x$ (dashed line), both methods yield similar results.

SUPPLEMENTARY NOTE 3: STATISTICS OF THE TRAJECTORIES

For all experimental tracked trajectories, the procedure described above for obtaining the parameters was applied. Supplementary Fig. 3(a) shows a scatter plot of the mean speed $\bar{v}$ and the torque Ω obtained for each bacterium. Additionally, histograms of these parameters are shown. The average speed over the entire bacterial population is $\langle \bar{v} \rangle = 39.45\,\mu\mathrm{m\,s^{-1}}$, which agrees well with the average speed of $43.2\,\mu\mathrm{m\,s^{-1}}$ for *Salmonella* Typhimurium (ST) which has been reported in the literature [6]. Averaging the effective torque Ω over the whole population gives a value of $\langle \Omega \rangle = 0.5\,\mathrm{s^{-1}}$, from which an average radius of the circular trajectories can be calculated by $\langle R \rangle \approx \langle \bar{v} \rangle / \langle \Omega \rangle \approx 80\mu$m. Given that to the best of our knowledge such measurements have not been performed in ST, we use as reference values those measurements performed with *E. coli*. For *E. coli*, radii between 10-50 μm have been reported, but note that *E. coli* generally swims at a speed of about 25 $\mu\mathrm{m\,s^{-1}}$, which leads to smaller circles [7].

Experimentally obtained rotational diffusion coefficients D_θ and parameter combinations (D_v/k_v^2) are displayed in Supplementary Fig. 3(b). The population average of the rotational diffusion coefficient gives $\langle D_\theta \rangle \approx 0.076$, which yields a reorientation time of the bacterial axis of about $\langle D_\theta \rangle^{-1} \approx 14$ s. This value is comparable to the reported $\langle D_\theta \rangle^{-1} \approx 17$ s to few minutes for *E. coli* bacteria in bulk [8–10], where again the comparison is performed with *E. coli* due to the lack of such measurements on ST. The population average over (D_v/k_v^2) yields $\langle D_v/k_v^2 \rangle = 2.38$. To the best of knowledge, no measurements of the strength of speed fluctuations in ST exist that can be compared to the results presented here.

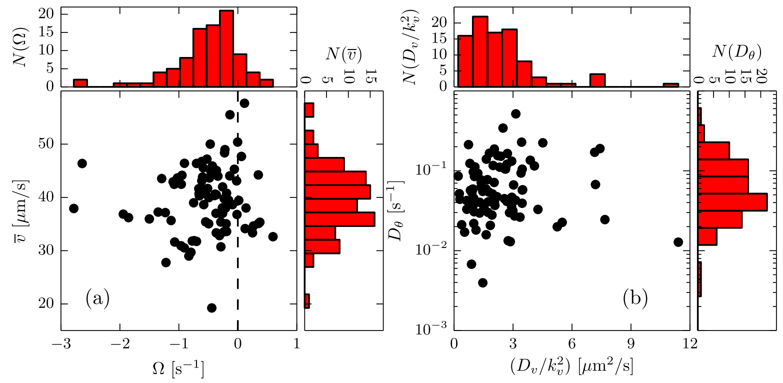

Supplementary Fig. 3: Parameters obtained from the experimental data for all trajectories (n=89, black dots). (a) The mean speed $\bar{v}$ as a function of Ω, and (b) the rotational diffusion coefficients D_θ as a function of the parameter combination D_v/k_v^2 for the experimental data. Also shown are histograms for each parameter: N represent the number of occurrences of Ω [top (a)], $\bar{v}$ [right (a)], D_v/k_v^2 [top (b)] and D_θ [right (b)].

SUPPLEMENTARY NOTE 4: ORIGIN OF FLUCTUATIONS – PROBING THERMAL FLUCTUATIONS

The Langevin equations for a particle undergoing solely thermal Brownian motion can be expressed as

$$\dot{\mathbf{x}}(t) = \sqrt{2D_\parallel}\xi_\parallel(t)\mathbf{e}(\theta) + \sqrt{2D_\perp}\xi_\perp(t)\mathbf{e}_\perp(\theta),$$
$$\dot{\theta}(t) = \sqrt{2D_\theta}\xi_\theta(t). \tag{26}$$

The strength of thermal noise is characterized by three parameters: $D_\parallel$ and $D_\perp$ in units of μm^2/s are the parallel and perpendicular components of the diffusion tensor, and D_θ in units of 1/s describes the thermal rotational reorientation of the long axis of the particle. Due to their thermal origin, they can generally be expressed via the Einstein relation:

$$D = \frac{k_B T}{\gamma}. \tag{27}$$

Here, γ is the drag friction coefficient, $k_B = 1.38 \cdot 10^{-23}$ J/K the Boltzmann constant and T the temperature. For obtaining the diffusion coefficients, the rotational friction coefficient γ_φ and the translational friction coefficients $\gamma_\parallel$

and $\gamma_\perp$ parallel and perpendicular to the particle's long axis have to be inserted in the former equation. Two well-established models exist for which analytical expressions for γ can be derived. The models approximate the particle as an ellipsoid (E) or as a sequence of interconnected spheres (Shish-Kebab model, SK). For details, see [11, 12]. The friction coefficients depend on the particle's shape properties, namely its length L, width b, aspect ratio ω and volume V as well as on the surrounding fluid's viscosity η. The analytical derivations for these two models yield for the friction coefficients

$$\gamma_\varphi^{\mathrm{E}} = \frac{4\eta V\left(\omega^4-1\right)}{\omega\left[\frac{2\omega^2-1}{\sqrt{\omega^2-1}}\log(\omega+\sqrt{\omega^2-1})-\omega\right]}, \tag{28}$$

$$\gamma_\parallel^{\mathrm{E}} = \frac{8\pi\eta b}{\left[\frac{2\omega}{1-\omega^2}+\frac{2\omega^2-1}{(\omega^2-1)^{3/2}}\log\left(\frac{\omega+\sqrt{\omega^2-1}}{\omega-\sqrt{\omega^2-1}}\right)\right]}, \tag{29}$$

$$\gamma_\perp^{\mathrm{E}} = \frac{8\pi\eta b}{\left[\frac{\omega}{\omega^2-1}+\frac{2\omega^2-3}{(\omega^2-1)^{3/2}}\log(\omega+\sqrt{\omega^2-1})\right]}, \tag{30}$$

for the ellipsoid model [11], where the ellipsoids volume is given by $V = (4/3)\pi L b^2$ and

$$\gamma_\varphi^{\mathrm{SK}} = \frac{\pi\eta L^3}{4}, \tag{31}$$

$$\gamma_\parallel^{\mathrm{SK}} = \frac{2\pi\eta L}{\log(\omega)}, \tag{32}$$

$$\gamma_\perp^{\mathrm{SK}} = 2\gamma_\parallel^{\mathrm{SK}}, \tag{33}$$

for the Shish-Kebab model [12]. Inserting these friction coefficients in Supplementary Eq. (27) give the rotational and translational diffusion coefficients for both the Shish-Kebab model, D_φ^{SK}, $D_\parallel^{\mathrm{SK}}$ and $D_\perp^{\mathrm{SK}}$, and the ellipsoid model, D_φ^{E}, $D_\parallel^{\mathrm{E}}$ and $D_\perp^{\mathrm{E}}$.

As all three diffusion coefficients are not explicit parameters of the model in Eqs. (3), a meaningful way of comparing with the model parameters has to be established. Therefore, a model for an active rod-shaped particle with orientation φ, moving at constant speed $\bar{v}$, undergoing a torque Ω and experiencing thermal fluctuations is considered:

$$\dot{\mathbf{x}}(t) = \left(\bar{v}+\sqrt{2D_\parallel}\xi_\parallel(t)\right)\mathbf{e}(\varphi(t)) + \sqrt{2D_\perp}\xi_\perp(t)\mathbf{e}_\perp(\varphi(t)), \tag{34}$$

$$\dot{\varphi}(t) = \Omega + \sqrt{2D_\varphi}\xi_\varphi(t). \tag{35}$$

In this model a propelling force acts along the long axis of a rod-shaped object. Due to this arrangement, the rotational diffusion coefficient D_φ influences both the particles orientation and the moving direction in the same way. Therefore, D_φ from the drag friction coefficient models can be directly compared to D_θ from the experimental data. The mean-squared displacement for the former mentioned model can be calculated:

$$\left\langle(\mathbf{x}(t)-\langle\mathbf{x}(t)\rangle)^2\right\rangle = 2(D_\parallel + D_\perp)t \qquad \text{for } t \ll D_\theta^{-1}, \Omega^{-1}. \tag{36}$$

For the model with correlated speed fluctuations, Eq. (3c), this quantity yields

$$\left\langle(\mathbf{x}(t)-\langle\mathbf{x}(t)\rangle)^2\right\rangle \approx 2\left(\frac{D_v}{k_v^2}\right)t + \text{const.} \quad \text{for } k_v^{-1} \ll t \ll D_\theta^{-1}, \Omega^{-1}. \tag{37}$$

Therefore, it is plausible to compare (D_v/k_v^2) from the data analysis with $D_T := (D_\parallel + D_\perp)$ from Shish-Kebab and ellipsoid model to check whether the observed fluctuations are of thermal origin. Thereby, the translational diffusion is considered as a whole. Diffusion parallel and perpendicular to the bacteria's long axis is not considered separately, as it is not resolved by the experiments at hand.

Bacterial lengths of 1.6 μm $< L <$ 10.1 μm and widths of 0.9 μm $< b <$ 2.2 μm are observed in the experiments. From these the corresponding aspect ratios ω can be calculated, which are displayed in Fig. 3(c). Using the temperature $T =$ 310 K at which the experiments were conducted, as well as the viscosity of water at this temperature $\eta = 0.69$ mPa s, the expected diffusion coefficients for both models are calculated for each bacterium individually. From these sets of

diffusion coefficients, the largest and smallest values for D_φ^{SK}, D_T^{SK}, D_φ^{E} and D_T^{E} define the ranges for both models as:

$$\begin{aligned} 0.0076 < \; & D_\varphi^{\mathrm{SK}} < 1.7, \\ 0.0017 < \; & D_\varphi^{\mathrm{E}} < 0.11, \\ 0.05 < \; & D_T^{\mathrm{SK}} < 0.59, \\ 0.41 < \; & D_T^{\mathrm{E}} < 1.06. \end{aligned}$$

Strictly speaking, these values rely on models for drag friction coefficients for unconfined motion in three-dimensional space. However, studies suggest that confinement due to nearby surfaces increases the drag friction coefficients which leads to smaller diffusion coefficients [11, 13]. Therefore, the calculated values can be seen as upper bounds for the diffusion coefficients.

SUPPLEMENTARY NOTE 5: BIASED-MOTION TESTS

Here we study how the presence of cells affects bacterial motility. We designed a statistical method that allows us to identify if bacteria are re-directing their motion towards or away from the cells. The method works as follows: For each trajectory, we measure the distance to the closest cell, l_{min}, as a function of time. From the sign of the derivative of this quantity it is possible to know if the trajectory is approaching or leaving the closest cell. Then, we take an average of the time derivative of l_{min}, that we call the bias index, $\overline{m}$. $\overline{m}$ tell us if on average the trajectory moved closer or away from the cells. In Supplementary Fig. 4 we show an example of one trajectory (panel (a)), together with its respective value of l_{min} as a function of time (panel (b)) and its time derivative (panel (c)). From the example, we can appreciate that the trajectory approaches and move away from the cells along the time, giving a $\overline{m}$ value close to zero. Naturally, $\overline{m}$ by itself it is not enough to conclude that the bacterium presents some degree of attraction or repulsion by the presence of cells, as any trajectory that does not feel any influence by the cells would anyway approach or move away randomly from cells. In order to derive some conclusion, we need to see what happens at the level of the whole population. In Supplementary Fig. 5 we show l_{min} for all the trajectories of ST in the presence of HC together with their respective time derivative. In panel (a) we can observe that some trajectories approach the cells while others move away from them. From panel (b) we observe that in general, the value of dl_{min}/dt fluctuates around zero, showing that as a population no preferential tendency is observed. In Supplementary Fig. 5(c) we show the histogram of $\overline{m}$ obtained from the previous trajectories. We observe a symmetric distribution centred around zero, confirming that as a population no bias towards cell is observed.

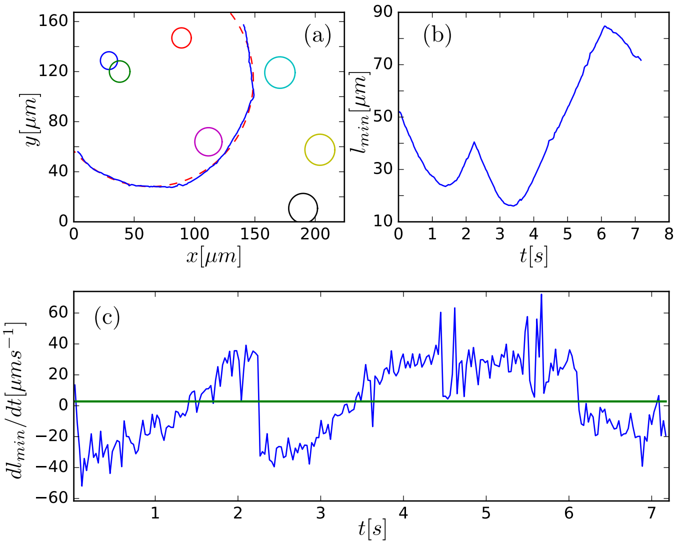

Supplementary Fig. 4: Illustration of the biased-motion test. (a) An experimental trajectory of ST (blue line) and its fitted trajectory (red dashed line). Color circles represent the T84 cells in the experiment. (b) Distance to the closest cell, l_{min}, as a function of time for the trajectory and the cells showed in (a). (c) Time derivative of l_{min} as a function of time. The sign indicates if bacteria move towards (positive) or away (negative) the closest cell. The green line indicates the time average, that we call $\overline{m}$.

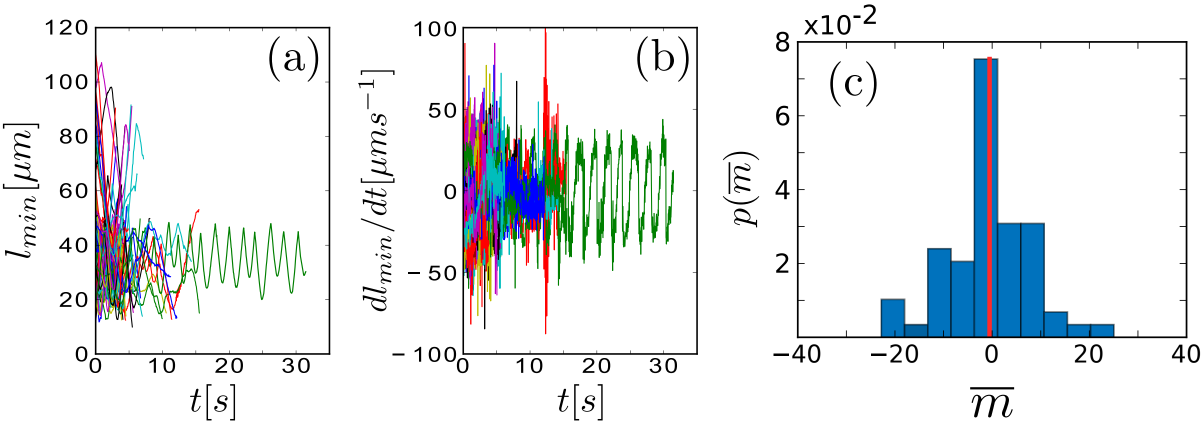

Supplementary Fig. 5: Statistical analysis of the biased-motion test. Distance to the closest cell (a) and its time derivative (b) as a function of time for series of experimental trajectories of ST in the presence of HC (colored lines). From each curve in (b) we take the average and compute the bias index $\overline{m}$. (c) Histogram of $\overline{m}$. The red line shows the average of all the $\overline{m}$.

We also test a different method to indicate if there was a bias towards cells, that consisted in measuring the curvature of the trajectories, κ as indicated in Supplementary Eq. (24). Similarly to the distance to the closest cell method, here we study how the curvature, κ, changes with time. The idea is that if cells exert an attraction (or repulsion) to bacteria, the curvature of trajectories should change as approaching to cells. In that sense, one would expect a correlation between the curvature κ and the distance to cells, l. In Supplementary Fig. 6 we show an example of a trajectory of ST in the presence of cells and also the measured curvature, κ, as a function of time. Panel (c) shows a plot of κ versus the distance to each cell, l. We do not observe any clear correlation between κ and l.

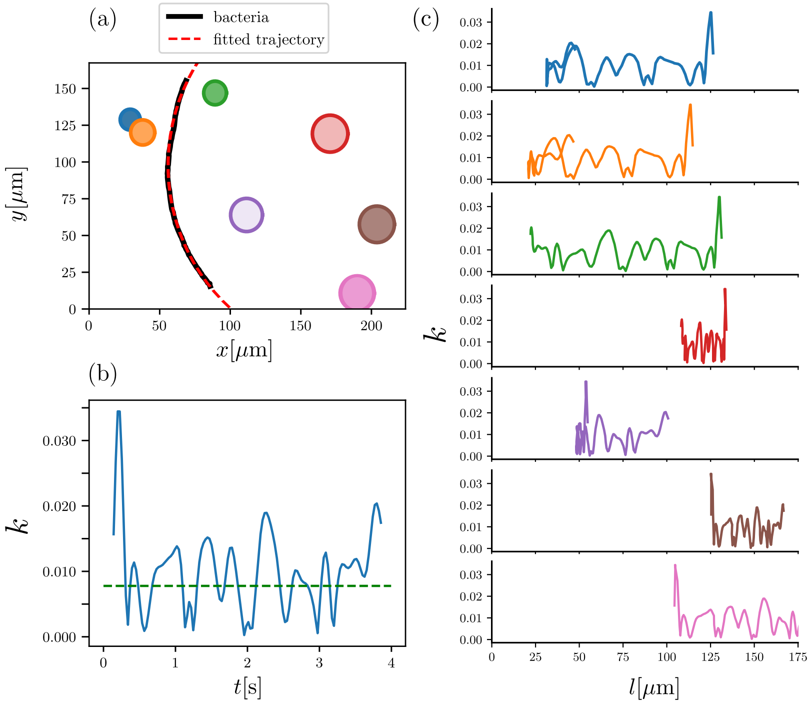

Supplementary Fig. 6: Correlation between the curvature κ and distance l to cells. (a) As in Supplementary Fig. 4, an example of an experimental trajectory of ST (black line) in the presence of host cells, represented in colored circles. (b) Curvature of trajectory, κ, as a function of time. The green dashed–line is the average curvature. (c) κ as a function of the distance to each cell in panel (a). The color of each curve corresponds to the distance to the cell marked by the same color. Curvature does not change with the distance to the cells.

Biased motion simulations

In order to test the method to detect biased motion, we performed simulations using bacteria that feel attraction towards cells. For that, we introduce an attractive field that re-orientate bacteria towards cells as following:

$$\dot{\theta}(t) = \Omega + \sum_j \frac{I_0}{d_j} \sin(\phi_j - \theta) + \sqrt{2D_\theta}\xi_\theta(t), \quad (38)$$

where j is an index that identifies each cell, I_0 is the intensity of the field and ϕ_j and d_j are the direction and the distance between the bacterium and the cell j. Each cell exerts an attraction to the bacteria: bacteria redirect their direction of motion towards the cells. The attraction is stronger as the bacterium gets closer to a cell.

SUPPLEMENTARY NOTE 6: COMPUTING SEARCH TIMES

When the characteristic distance between HCs, ${\rho_{HC}}^{-1}$, is much larger than $\bar{v}\Omega^{-1}$, we can expect mean first passage time $\langle\tau\rangle$ to be mainly determined by the diffusion coefficient D such that $\langle\tau\rangle \propto D^{-1}$; otherwise, the computation of the average time of encounters between bacteria and HCs should be computed in a ballistic regime. All this means that for a fixed density ρ_{HC}, $\langle\tau\rangle$ strongly depends on Ω. This is illustrated in Supplementary Fig. 7. This is also observed in experiments as shown in Fig. 5 of the main text.

To ensure that the fist passage time statistics was identical to the one expected for unbiased, random encounters, we measured the first passage time in experiments without HCs by using what we called "ghost cells" (GC) (see Fig. 5 (a) in the main text). The obtained results provide additional support for (i) absence of chemotaxis towards HCs, and (ii) that motility parameter remain unchanged in the presence of HCs. We proceed as follows. We computed the time it takes for ST-WT to find particular regions in the space of size and distribution comparable to the one of HCs; we refer to these regions as "ghost cells" (GC). To be specific, we took the trajectories of ST-WT in absence of HC and computed τ as the time it takes for the trajectory to reach one GC. GC were distributed as in experiments with ST-WT in presence of HC, as we show in Supplementary Fig. 8. The mean values of the search times are $\langle\tau\rangle_{ST+HC} = 3.3s \pm 2.8s$, $\langle\tau\rangle_{ST+GC} = 3.1s \pm 2.3s$ and $\langle\tau\rangle_{STM935+HC} = 3.5s \pm 2.4s$. These results, again, support the statement that there is no effect of the presence of HC in the ST motility and that HC act as simple obstacles.

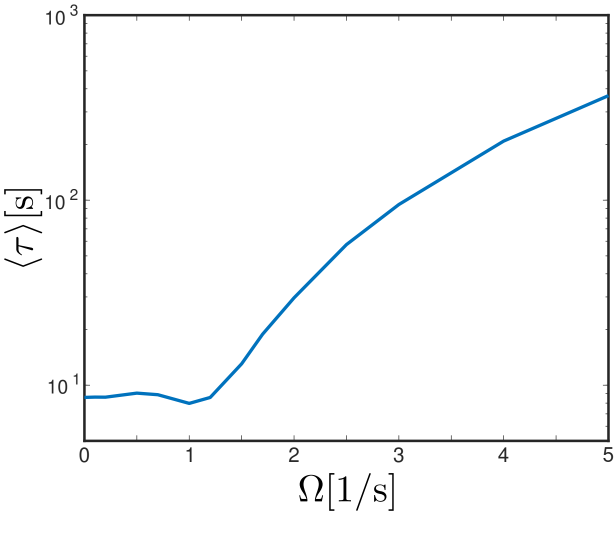

Supplementary Fig. 7: Mean first passage time $\langle\tau\rangle$ as a function of the angular speed Ω computed in simulations using the parameters given in Supplementary Table II, which correspond to average motility parameters obtained in the experiments with ST-WT.

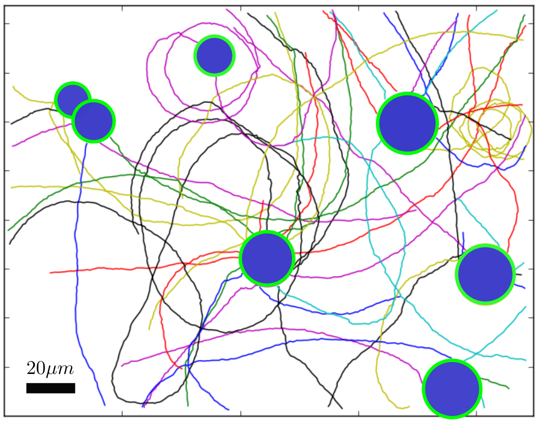

Supplementary Fig. 8: Search with "ghost cells" (GC). Experimental trajectories of ST-WT in absence of HC. Circular GC were located in space at densities and sizes as in experiments in Fig. 5 (a). Each color line represent a bacterial trajectory tracked from the experiment of ST-WT in the absence of HC. Trajectories are drawn until they encounter a GC. Search times between bacteria and the GC were computed considering trajectories from their beginning until they hit a GC.

SUPPLEMENTARY NOTE 7: CHARACTERIZATION OF HOST-CELLS SIZE, DISTRIBUTION AND DENSITY

Size and distribution of HC were analyzed using automatic object detection tools from fiji software [14]. The area occupied by each cell was measured leading to an average area of $A_{cells} = 395\mu m^2 \pm 150\mu m^2$. Since most cells showed a round shape, we considered then as circles with an average radius $R_{cells} = 10.97\mu m \pm 2.46\mu m$, see Supplementary Fig. 9 (a). Then, in order to measure the distribution and density we computed the distance between cells. In Supplementary Fig. 9 (b) we show the distribution of distances to the closest cell. Notice that the distribution is consistent with cells randomly placed over the space. From the mean value we estimate the inter-distance between cells, $d_{cells} = 55\mu m \pm 7\mu m$. Finally, considering circular cells randomly placed in space, we computed the density of cells, $\rho_{HC} \approx 1.69\,10^{-4}\mu\text{m}^{-2}$.

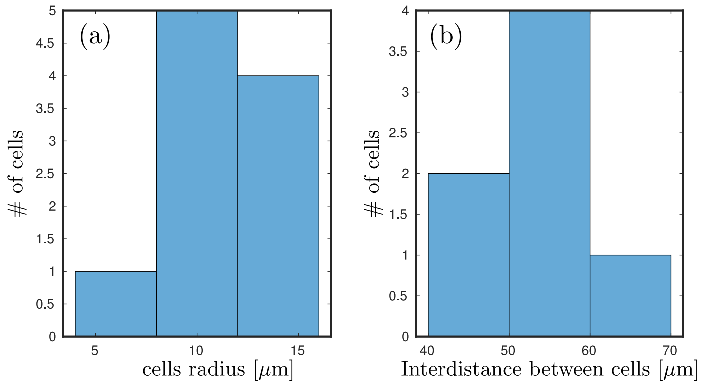

Supplementary Fig. 9: (a) Histogram of cells radius. Each cell was approximated by a circle of radius R. (b) Histogram of inter-distance between cells, from which ρ_{HC} was computed.

SUPPLEMENTARY NOTE 8: SIMULATIONS OF THE INFECTION PROCESS

We performed numerical simulations of bacteria moving over a surface where HC were distributed mimicking the size and distribution observed in experiments. Bacterial motility was integrated using Euler-Maruyama method according to Eqs. (3), using the parameters extracted from the experiments and showed in Supplementary Table II. Size and distribution of HC were also extracted from the experiment, see Supplementary Fig. 9. We computed the number of bacteria invading cells (NIB). We consider that whenever a bacterium reaches a cell, it can invade it with a probability

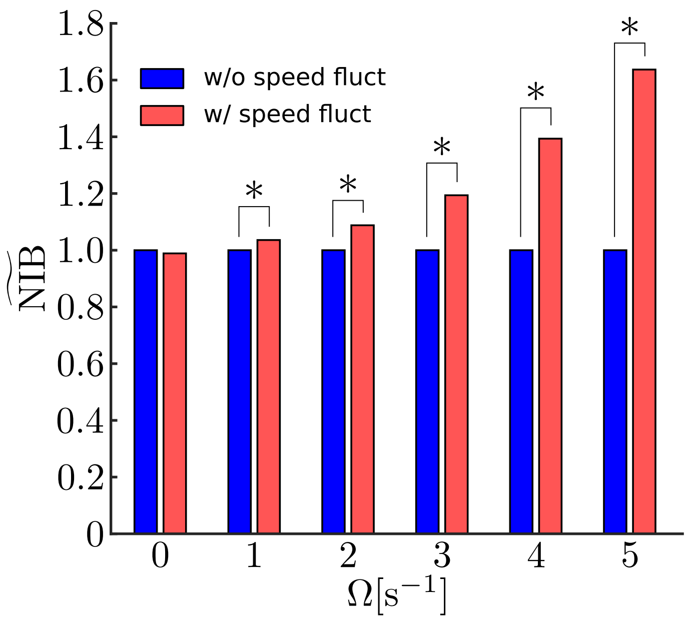

Supplementary Fig. 10: Assessment of the role of speed fluctuation in the infection process. Normalized average number of bacteria invading cells ($\widetilde{\text{NIB}}$) as function of Ω for simulations without (w/o speed fluct) and with speed fluctuations (w/speed fluct); for each value of Ω the normalization is performed with respect to the result obtained in simulations without speed fluctuations. One side Z-tests were performed to test against the alternative hypothesis that the mean of the NIB is larger in the presence of speed fluctuations. The asterisk ($*$) indicates p–value$<$ 0.01. Test details are in Supplementary Table I. Parameters as indicated in Supplementary Note 8 and Supplementary Table II.

p_s (otherwise, it keeps on exploring the space). If the invasion was successful, we increase in one unit NIB and re-introduce the bacterium in a new random position, in order to keep a constant density. As the NIB depends on the density of bacteria, we use a density of $\rho_{bact} = 7.8\,10^{-2}\mu\text{m}^{-2}$ that was adjusted from the experiments. Simulations were integrated using a time step $\Delta t = 0.01$s until a fixed time $T = 3600$s and the motility parameters provided in Supplementary Table II. Simulations in panel (g) in Fig. 5 were done using $p_s = 1.8\,10^{-4}$, which was fitted from invasion experiments reported in [6]. In the case of the mutants ST-M913, simulations were performed using Eqs. (26) and the same p_s value. The diffusion tensor was computed using the SK model.

To assess of the role of speed fluctuations in the infection process, we performed simulations of the infection model using the parameters indicated above and varied the value of Ω for $T = 1000$s. Supplementary Fig. 10 shows the normalized average number of bacteria invading cells (NIB*) as function of Ω for simulations without speed fluctuations and with speed fluctuations. For values of $\Omega \geq 1$, speed fluctuations lead to statistical significant increments of NIB that are larger than 15% for $\Omega \geq 3\text{s}^{-1}$. Simulations were implemented with a custom code in C++ and using the GNU Scientific Library (GSL) (version gsl-2.3).

Supplementary Table I: Statistical tests.

Test	Null hypothesis	par/dist	p–value
Fig. 4(c): Two–sided independent two-samples t–test.	ST-WT & ST-WT + T84 same distribution	$\overline{v}$	4.34×10^{-12}
		Ω	6.09×10^{-3}
		D_θ	0.52
		D_v/k_v^2	6.56×10^{-10}
	ST-WT & ST-M935 + T84 same distribution	$\overline{v}$	0.06
		Ω	0.18
		D_θ	0.07
		D_v/k_v^2	0.29
Fig. 4(e)–(f): Two–sided binomial test	p(sgn($\overline{m}$))=0.5	ST-WT	0.60
		ST-M935	0.99
		SB-NB	0.92
		SB-B	2.24×10^{-54}
Supplementary Fig. 10: One–sided Z–tests	NIB w/ speed fluctuations $\leq$ NIB w/o speed fluctuations	$\Omega = 0\text{s}^{-1}$	0.80
		$\Omega = 1\text{s}^{-1}$	0.002
		$\Omega = 2\text{s}^{-1}$	1.35×10^{-4}
		$\Omega = 3\text{s}^{-1}$	2.92×10^{-6}
		$\Omega = 4\text{s}^{-1}$	3.98×10^{-10}
		$\Omega = 5\text{s}^{-1}$	6.50×10^{-13}

Supplementary Table II: Simulation parameters.

Parameters common to all simulations	
$\overline{v}$	39.45 μms^{-1}
Ω	0.5s^{-1}
D_θ	0.076s^{-1}
D_v	$7.2\,10^5$ μm^{2}s^{-3}
k_v	550s^{-1}
ρ_{HC}	$1.69\,10^{-4}\mu$m^{-2}
Δt	0.01s
Biased motion simulations	
I_0	1000μms^{-1}
Search time simulations	
N_{bact}	10^4
Infection process with and without speed fluctuations simulations	
Ω	{0,1, 2, 2.5, 3, 4, 5}s^{-1}
D_v	{0, $7.2\,10^5$} μm^{2}s^{-3}
k_v	{0, 550}s^{-1}
ρ_{bact}	$7.8\,10^{-2}\mu$m^{-2}
p_s	1
T	1000s

SUPPLEMENTARY REFERENCES

[1] Gardiner, C. W. *Handbook of stochastic methods*, vol. 4 (Springer Berlin, 1985).

[2] Schienbein, M. & Gruler, H. Langevin equation, fokker-planck equation and cell migration. *B. Math. Biol.* **55**, 585–608 (1993).

[3] Press, W. H. *et al.* *Numerical recipes 3rd edition: The art of scientific computing* (Cambridge university press, 2007).

[4] Jones, E., Oliphant, T., Peterson, P. *et al.* SciPy: Open source scientific tools for Python (2001–). URL `http://www.scipy.org/`. [Online; accessed 2016-05-04].

[5] Tinevez, J.-Y. *et al.* Trackmate: An open and extensible platform for single-particle tracking. *Methods* **115**, 80–90 (2017).

[6] Pontier-Bres, R. *et al.* Modification of salmonella typhimurium motility by the probiotic yeast strain saccharomyces boulardii. *PloS one* **7** (2012).

[7] Lauga, E., DiLuzio, W. R., Whitesides, G. M. & Stone, H. A. Swimming in circles: motion of bacteria near solid boundaries. *Biophys. J.* **90**, 400–412 (2006).

[8] Lauga, E. & Powers, T. R. The hydrodynamics of swimming microorganisms. *Rep. Prog. Phys.* **72**, 096601 (2009).

[9] Berg, H. C. & Brown, D. A. Chemotaxis in escherichia coli analysed by three-dimensional tracking. *Nature* **239**, 500–504 (1972).

[10] Drescher, K., Dunkel, J., Cisneros, L. H., Ganguly, S. & Goldstein, R. E. Fluid dynamics and noise in bacterial cell–cell and cell–surface scattering. *Proceedings of the National Academy of Sciences* **108**, 10940–10945 (2011).

[11] Han, Y., Alsayed, A., Nobili, M. & Yodh, A. G. Quasi-two-dimensional diffusion of single ellipsoids: Aspect ratio and confinement effects. *Phys. Rev. E* **80**, 011403 (2009).

[12] Doi, M. & Edwards, S. F. *The theory of polymer dynamics*, vol. 73 (oxford university press, 1988).

[13] Levine, A. J., Liverpool, T. & MacKintosh, F. Mobility of extended bodies in viscous films and membranes. *Phys. Rev. E* **69**, 021503 (2004).

[14] Schindelin, J. *et al.* Fiji: an open-source platform for biological-image analysis. *Nature methods* **9**, 676–682 (2012).
