# Supplementary material for: Statistics of pathogenic bacteria in the search of host cells
Source: Nat Commun. 2021 Mar 31;12:1990. doi: 10.1038/s41467-021-22156-6 (PMC8012381; doi:10.1038/s41467-021-22156-6)
Supplement: Supplementary file 3 — Description of Additional Supplementary Files [file 41467_2021_22156_MOESM3_ESM.pdf]

Description of Additional Supplementary Files

File name: Supplementary Movie 1
Description: ST motion close to a surface, highlighting a bacterium with clockwise motion.

File name: Supplementary Movie 2
Description: ST motion close to a surface, highlighting a bacterium with straight motion.

File name: Supplementary Movie 3
Description: ST motion close to a surface, highlighting a bacterium with counterclockwise motion.

File name: Supplementary Movie 4
Description: Same bacterial motion as shown in Supplementary Movie 1, but with the temporal evolution of the speed shown in a separate plot.

File name: Supplementary Movie 5
Description: ST swimming in the presence of human T84 epithelial colonic cells. Notice the variability in motility patterns and how bacteria moving in circles are less efficient in finding host cells.
