# Supplementary figures and images for: Statistics of pathogenic bacteria in the search of host cells
Source: Nat Commun. 2021 Mar 31;12:1990. doi: 10.1038/s41467-021-22156-6 (PMC8012381; doi:10.1038/s41467-021-22156-6)

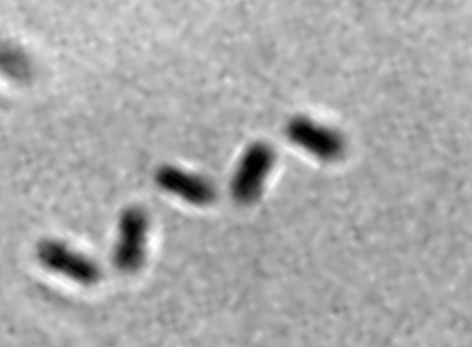

Supplement: Supplementary file 9 — Source Data [file 41467_2021_22156_MOESM9_ESM.zip › Fig1/TimeSeries2.png]

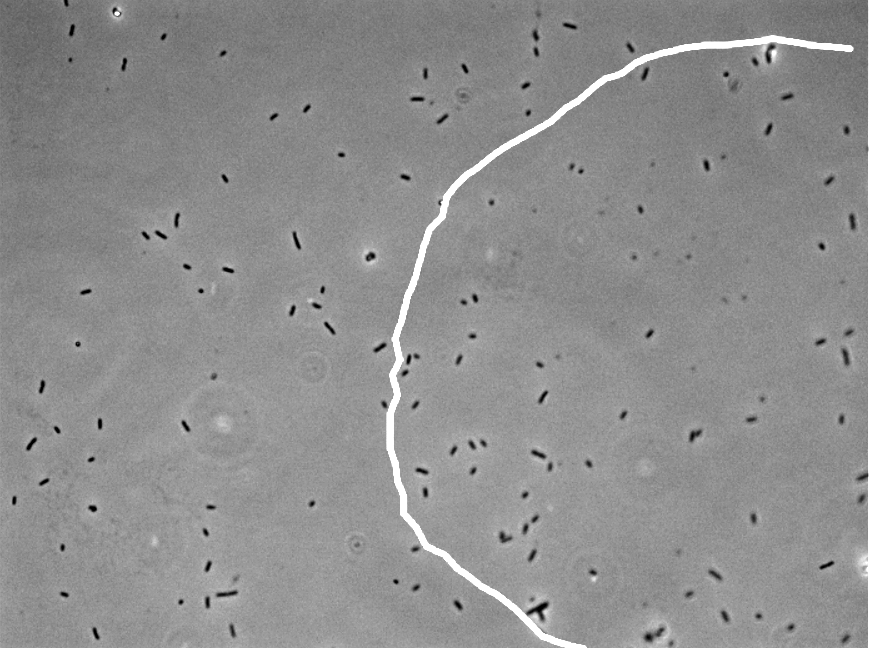

Supplement: Supplementary file 9 — Source Data [file 41467_2021_22156_MOESM9_ESM.zip › Fig1/imagej_CCW_enhanced.png]

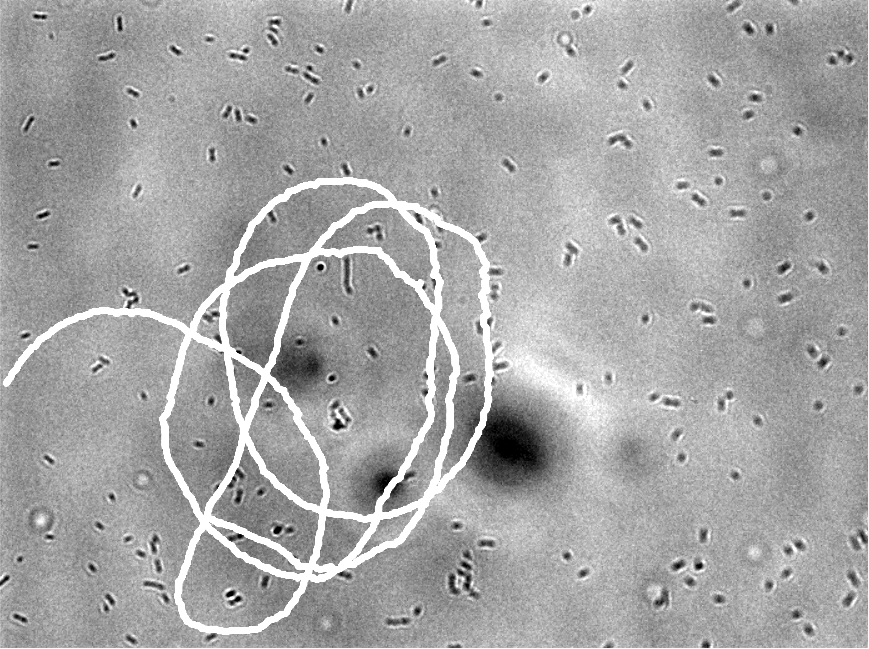

Supplement: Supplementary file 9 — Source Data [file 41467_2021_22156_MOESM9_ESM.zip › Fig1/imagej_CW_enhanced.png]

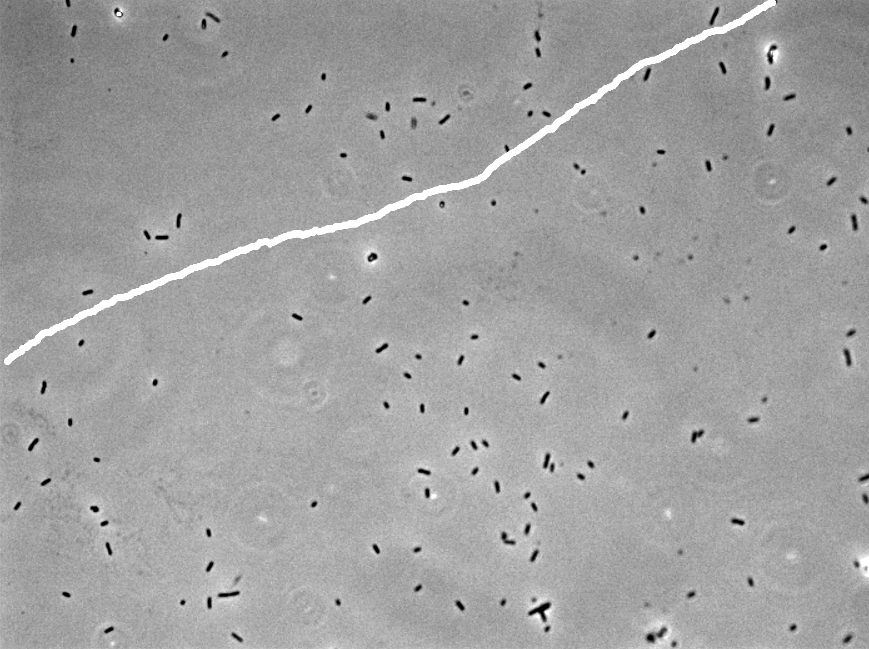

Supplement: Supplementary file 9 — Source Data [file 41467_2021_22156_MOESM9_ESM.zip › Fig1/imagej_straight_enhanced.png]

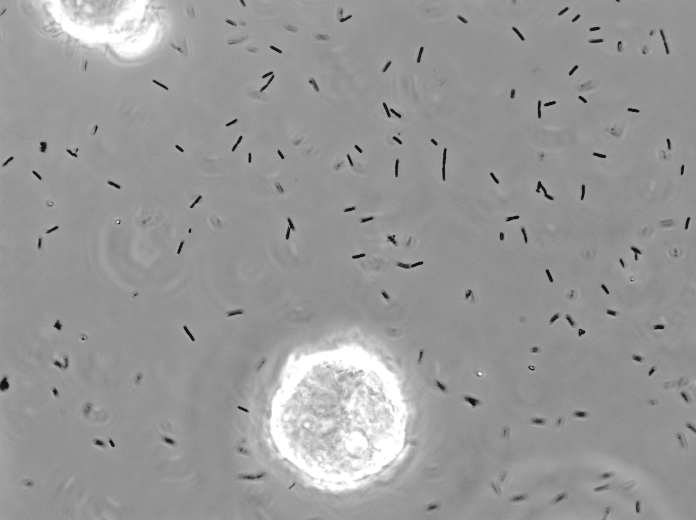

Supplement: Supplementary file 9 — Source Data [file 41467_2021_22156_MOESM9_ESM.zip › Fig4/ST-M935_&&_T84_cells.png]

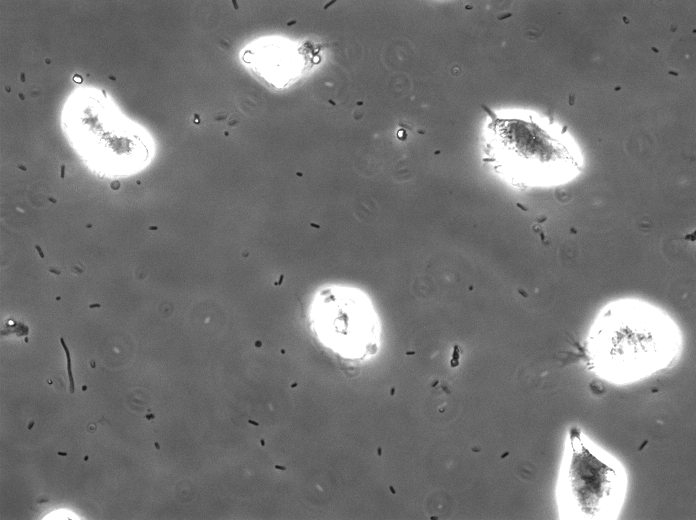

Supplement: Supplementary file 9 — Source Data [file 41467_2021_22156_MOESM9_ESM.zip › Fig4/ST_WT_&&_T84_cells.png]
